# Advances in epidermal growth factor receptor specific immunotherapy: lessons to be learned from armed antibodies

**DOI:** 10.18632/oncotarget.27730

**Published:** 2020-09-22

**Authors:** Fleury Augustin Nsole Biteghe, Neelakshi Mungra, Nyangone Ekome Toung Chalomie, Jean De La Croix Ndong, Jean Engohang-Ndong, Guillaume Vignaux, Eden Padayachee, Krupa Naran, Stefan Barth

**Affiliations:** ^1^Department of Radiation Oncology and Biomedical Sciences, Cedars-Sinai Medical, Los Angeles, CA, USA; ^2^Medical Biotechnology & Immunotherapy Research Unit, Institute of Infectious Disease and Molecular Medicine, Faculty of Health Sciences, University of Cape Town, Cape Town, South Africa; ^3^South African Research Chair in Cancer Biotechnology, Department of Integrative Biomedical Sciences, Faculty of Health Sciences, University of Cape Town, Cape Town, South Africa; ^4^Sun Yat-Sen University, Zhongshan Medical School, Guangzhou, China; ^5^Department of Orthopedic Surgery, New York University School of Medicine, New York, NY, USA; ^6^Department of Biological Sciences, Kent State University at Tuscarawas, New Philadelphia, OH, USA; ^7^Arctic Slope Regional Corporation Federal, Beltsville, MD, USA; ^8^Department of Physiology, University of Kentucky, Lexington, KY, USA; ^*^These authors contributed equally to this work

**Keywords:** epidermal growth factor receptor (EGFR), recombinant immunotoxins (ITs), targeted human cytolytic fusion proteins (hCFPs), recombinant antibody-drug conjugates (rADCs), recombinant antibody photoimmunoconjugates (rAPCs)

## Abstract

The epidermal growth factor receptor (EGFR) has been recognized as an important therapeutic target in oncology. It is commonly overexpressed in a variety of solid tumors and is critically involved in cell survival, proliferation, metastasis, and angiogenesis. This multi-dimensional role of EGFR in the progression and aggressiveness of cancer, has evolved from conventional to more targeted therapeutic approaches. With the advent of hybridoma technology and phage display techniques, the first anti-EGFR monoclonal antibodies (mAbs) (Cetuximab and Panitumumab) were developed. Due to major limitations including host immune reactions and poor tumor penetration, these antibodies were modified and used as guiding mechanisms for the specific delivery of readily available chemotherapeutic agents or plants/bacterial toxins, giving rise to antibody-drug conjugates (ADCs) and immunotoxins (ITs), respectively. Continued refinement of ITs led to deimmunization strategies based on depletion of B and T-cell epitopes or substitution of non-human toxins leading to a growing repertoire of human enzymes capable of inducing cell death. Similarly, the modification of classical ADCs has resulted in the first, fully recombinant versions. In this review, we discuss significant advancements in EGFR-targeting immunoconjugates, including ITs and recombinant photoactivable ADCs, which serve as a blueprint for further developments in the evolving domain of cancer immunotherapy.

## INTRODUCTION

Epidermal growth factor receptor (EGFR) belongs to a family of transmembrane proteins that are known as tyrosine kinases (ErbB family) and made of four members known as: EGFR/HER1, ErbB2/HER2, ErbB3/HER3, and ErbB4/HER4 [1–4]. EGFR is a 170 kDa glycoprotein, known as HER1 or c-ErbB-1, and was the first member of receptor tyrosine kinase (RTK) to be described [4]. EGFR is characterized by an extracellular ligand binding domain (ectodomain), a single transmembrane domain (TM), and an intracellular domain with tyrosine function [1–3]. EGFR activation begins with ligand binding induced ectodomain dimerization (homo- and/or heterodimerization), causing the autotransphosphorylation of tyrosine residues located on the intracellular tyrosine kinase domain [3, 4]. This tyrosinase phosphorylation consequently recruits signal transducers and activators of intracellular substrates such as Rat sarcoma (Ras). Once activated, Ras activates downstream signaling cascades such as RAF/MEK1/2/ERK1/2, and/or PI3k/Akt, regulating cell proliferation, survival, differentiation, and migration [1, 2, 5]. The EGFR signaling pathway is tightly regulated under normal conditions. However, EGFR has been aberrantly expressed in many cancers due to mutations associated with poor cancer prognosis [4, 6–12]. Hence, developing new therapeutic approaches that target EGFR, becomes very pertinent.

So far, two EGFR-targeted therapeutic approaches have been developed using antagonist monoclonal antibodies (mAbs) or small molecule tyrosine kinase inhibitors (TKIs), either blocking ligand binding or inhibiting tyrosinase function by preventing adenosine triphosphate (ATP) binding to the EGFR intracellular domain [13]. Both therapeutic strategies have been clinically approved for treating multiple cancers [12–14]. However, compromised efficacy of TKIs is associated with increased mutations in tyrosine kinase intracellular domains. These mutations were found to drive resistance to TKIs by increasing ATP avidity to the targeted domain or by constitutively activating downstream signaling pathways causing treatment failure [15–18]. Conversely to TKIs, mAbs partly exert their cytotoxic effects by reducing EGFR ectodomain density through induction of receptor mediated endocytosis or by activating antibody-dependent cellular cytotoxicity (ADCC) towards EGFR positive cancer cells [19–21]. To date, five mAbs have been clinically approved and target different ErbB family members: EGFR (HER1): cetuximab (2004, head and neck and colorectal cancers), panitumumab (2006, colorectal cancer), necitumumab (2015, non-small lung cancer); and HER2: trastuzumab (1998, breast cancer) and pertuzumab (2012, breast cancer) [14, 19–26]. Although promising, these naked antibody-based monotherapies have achieved poorer clinical responses, than when combined with conventional chemotherapy, radiotherapy or TKIs [23]. Despite obvious clinical benefits, these combination therapies were associated with undesirable side effects, partly owing to mAbs bulky size limiting tumor penetration or rodent origin, inducing an immune response when used in immunocompetent patients [27, 28]. Consequently, mAbs were considered to be armed with cytotoxic drugs to generate so called antibody-drug conjugates (ADCs) which could tilt the toxicity/therapeutic balance towards a more beneficial specific therapeutic efficacy. These ADCs are able to achieve improved selective cytotoxicity based on their ability to discriminate and exploit the differential cell surface expression of tumor associated antigens (TAA) between diseased and healthy tissues, and use it as a mechanism to specifically deliver the conjugated cytotoxic payloads to the tumor site [29–31]. Major drawbacks of antibodies chemically conjugated to highly potent cytotoxic small molecule toxins are still related to immunogenicity but also toxin release causing off-target toxicities. Therefore, further ADC refinement should ideally produce immunoconjugates, which are non-immunogenic and non-toxic in their native administered state, with toxicity only unleashed when internalized into targeted tumor cells. Protein engineering was consequently allowing to replace small molecule toxins by cytotoxic proteins originally derived from plants and bacteria in so-called immunotoxins (ITs) and later by replacing these highly immunogenic protein toxins by human apoptosis inducing enzymes to generate targeted human cytolytic fusion proteins (hCFPs) for cancer therapy [32–34]. In spite of their initial preclinical promises, enzymes to be used for the generation of hCFPs might be blocked by the activity of their natural inhibitors upregulated in tumor cells to allow escape from immune responses [32, 34, 35]. To date, preclinical proofs of concept have been described for hCFPs with improved rationally designed inhibitor insensitive variants of the protease granzyme B and the RNase angiogenin in addition to other human cytoskeleton interfering proteins, such as the microtubule-associated protein tau, or death-associated protein kinases to treat various cancers [32, 33, 35–37]. Likewise, in order to reduce off target effects described for released small molecule toxins from ADCs, light sensitive antibody-photoconjugates (APCs) were developed by replacing the toxic compounds by light inducible photosensitizers (PSs) showing essentially no toxicity to normal/non-irradiated tissues, as they require an extra step of light activation to exert their phototoxicity [9, 11, 38–43].

Altogether, these recent biotechnological advances have expanded the repertoire of armed antibodies through the development of different forms of ADCs. Hence, this review aims to introduce selected antibody-based therapeutic approaches and corresponding key technologies, allowing to describe recent developments exemplified for EGFR-targeting immunotherapies, concurrently comparing the therapeutic efficacies of the different treatment modalities and conclude on future perspectives.

## BRIEF OVERVIEW OF EGFR-SPECIFIC IMMUNOTHERAPEUTICS

### The evolution of antibody-mediated therapeutics

Cancer treatment is traditionally founded on three approaches; surgery, radiation and chemotherapy, which have shown limited therapeutic benefits in patients with metastatic disease [43, 44]. Despite the significant advances in the development of systemic treatment over the years, the therapeutic usage of toxic agents is a two-edged sword potentially causing normal organ toxicities, thus restricting treatment to certain therapeutic dosages [9]. In light of this, novel palliative treatment approaches were urgently needed to specifically treat patients with refractory and metastatic disease. Although a full discussion is outside the scope of this review, it suffices to say that cancer immunotherapy—in the form of adoptive cell therapy (ACT)—is an alternative therapeutic option, using the patient’s own immune system to control and destroy tumor cells [45, 46]. This therapeutic modality relies on antigen recognition of tumor cells by antigen presenting cells (e.g., dendritic cell) or engineered cytotoxic T-lymphocytes (e.g., chimeric antigen receptor T-cells) to specifically recognize and induce tumor destruction in an antigen-dependent manner [44–50]. Despite their initial clinical success, immune cell-based therapies have been limited in treating solid tumors due to T-cell exhaustion or their incapacity to infiltrate tumors.

To target receptors on solid tumors, tumor-specific mAbs binding to oncogenic cell surface receptors were developed as a form of molecular targeted immunotherapeutic treatment. Traditionally, this naked antibody-based immunotherapy induces tumor destruction through ADCC, complement-dependent cytotoxicity (CDC) or receptor blockade [29, 51–53]. Demonstration of the therapeutic potential of mAbs was performed using cetuximab (anti-EGFR mAb), which successively induces EGFR-specific tumor destruction through receptor blockade, subsequently causing EGFR endocytosis and inhibition of intracellular tyrosine kinase function regulating downstream pro-tumorigenic signals [54, 55]. To achieve this therapeutic goal, cetuximab has shown to trigger apoptosis by impairing the cell cycle, reducing angiogenesis, tumor cell invasion, metastases and activating an antitumor immune response [56, 57]. The most notable clinical success using ErbB-mAbs leading to FDA approval was accomplished using trastuzumab (Herceptin, 1998, breast cancer targeting HER2) and cetuximab (Erbitux, targeting HER1 or EGFR) which significantly prolonged head and neck squamous cell carcinoma (HNSCC) patient survival (from 29.3 to 49 months) when combined with chemo- (Cisplatin or carboplatin and 5-Fluorouracil) and radiotherapy [58, 59]. Likewise, another anti-EGFR IgG2 mAb, panitumumab (Vectibix), was FDA approved (September 2006) as a first-line or palliative therapy (following chemotherapy using fluoropyrimidine, oxaliplatin and irinotecan) to treat metastatic colorectal cancer patients [14, 60, 61]. This regimen was clinically approved as it offered superior patient survival (96.4 vs 59.7 days), than the best supportive care treatment alone [14, 62]. Similarly, pertuzumab (2012: anti-HER2), and necitumumab (2015: anti-HER1) were FDA approved for treating HER2-positive breast and EGFR-positive non-small lung cancers (NSLCs), respectively [14, 19–26]. Despite their encouraging initial responses, their widespread application against tumor associated antigens (TAAs) was limited to a combination with immune checkpoint inhibitors only, as they did not offer significant therapeutic benefits against known preclinical animal xenograft models of human cancers [63–65]. Although simple enough in concept, their therapeutic application (mAbs) has been beset with multiple obstacles, owing to a combination of various factors including: (1) non-specific biomarker selection enabling the identification of irrelevant target tumor antigens; (2) inefficient potency of naked mAb as anticancer drugs; (3) poor tumor cell penetration of mAbs; (4) production of neutralizing antibodies (or anti-idiotypic antibody) against mAbs of human origin; and (5) off-target effects and immunogenicity when used in humans with functional immune systems, limiting repeated treatment dosage schedules [28, 66, 67]. These undesirable effects were reported to cause skin and cardiac toxicities, when treating colorectal and breast cancer patients with cetuximab, panitumumab and trastuzumab [62, 68, 69]. Collectively, these mAbs have the capacity to activate a human immune response able to neutralize administered human-mAbs (anti-idiotypic antibody) and alter their therapeutic efficacy [67, 70, 71]. The mitigation of these undesired effects, was rendered possible with the advent of DNA technology which led to the chimerization and humanization of antibodies able to reach clinical fruition [72].

### Chimeric, humanized, and generation of new Ab formats using recombinant DNA technology

**Figure 1:**
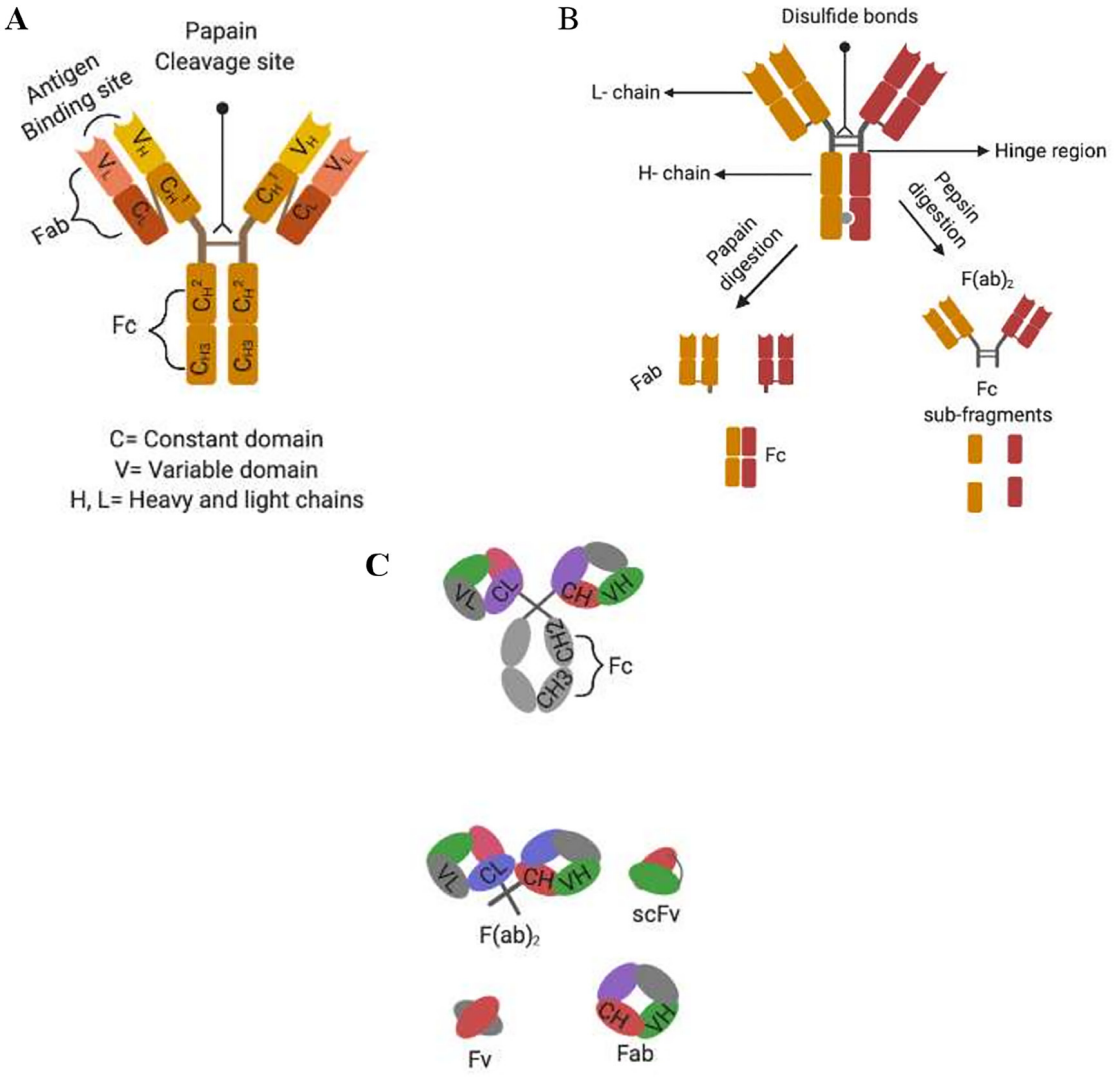
Alternative formats of engineered human antibodies. (**A**) Domain organisation of an IgG molecule; (**B**) separation of antibody function by proteolytic cleavage; (**C**) schematic representation of multivalent recombinant antibody constructs.

Monoclonal antibodies (mAbs) of the IgG isotype subtype are the most commonly used in immunotherapeutic treatment and are typically made of four polypeptides consisting of two heavy and light chains which are covalently linked together by disulphide bonds to form a “Y” structure (see Figure 1A). The tips of the heavy-light chain pairs form the antigen-binding domain (Fab) which is subdivided into seven amino acids, four of which are the framework regions (FRs) and three of which are the primary antigen recognition site known as complementarity-determining regions (CDRs) [9, 28]. On the other hand, each heavy chain is made up of three constant domains namely CH1, CH2, and CH3, as well as one variable domain (V_H_), while each light chain consists of one constant domain (C_L_) and one variable domain (V_L_) (Figure 1A). The antibody effector function is mediated by the fragment crystallizable (Fc) within the heavy chain constant region [52, 73]. The assembly of these domains is critical for normal antibody function. In light of this, antibody chimerization was developed with the intent to reduce mAbs immunogenicity, enable multiple dosing schedules and favoring assessment of their pharmacokinetic behavior and host immune activating function [74]. Chimerization is a transgenic manipulation consisting of fusing a murine-derived antibody variable region domain (Fab: antigen binding properties) with a human IgG constant region (Fc) possessing the effector functions that mediate ADCC (Figure 1A) [52]. Despite the clinical success and regulatory approval of these chimeric antibodies (e.g., Rituximab, FDA approved in 1997), they still possessed some human anti-mouse antibody (HAMA) responses [75]. Therefore, the next intuitive step in improving chimeric antibody properties led to humanization of the fragment variable regions (Fab) possessing antigen binding activity.

With the advent of recombinant DNA technology, mAb humanization consisting of grafting a murine CDR into a CDR depleted human IgG was performed. Using this grafting method, the anti-HER2 humanized antibody trastuzumab was produced and FDA approved in 1998 to treat HER2-positive cancers (breast, pancreas, and non-small cell of lung cancer) [76]. This humanized antibody was more potent than its murine counterpart and standard chemotherapy, based on its ability to efficiently activate ADCC [63, 77–81]. Other humanization procedures include veneering which relies on FRs manipulation [63, 77–81]. However this approach may seriously impair mAbs antigen binding capacity, which heavily relies on the topography and chemical structure of the CDRs and some FRs to maintain its binding affinity [78, 81]. These limitations paved the way to the development of transgenic mice, which enabled the production of fully human antibodies. These mice were engineered to possess functional human immunoglobulin transgenes, by replacing their mouse orthologues that were genetically inactivated [63, 80]. Once produced after immunization, such human mAb could be cloned and scaled up using hybridoma technology. For example, E7.6.3 mAb which specifically targeted EGFR was produced using this method [82]. During this study, E7.6.3 mAb was strongly binding to its cognate receptor and this binding was correlating with decreased cell viability and tumor eradication in mice, which showed no signs of recurrence up to 8 months after the last injection [82]. Nevertheless, human mAbs may acquire somatic mutations during their maturation process in transgenic mice within their FR and CDR regions [83]. Consequently these mAbs will no longer share complete sequence homology to their inherent human germline, which may predispose them to immunogenic reaction in humans [63, 83]. Because of these biotechnological advances, mAbs are starting to fulfill their therapeutic role as immunotherapeutic agents. Of late, antibody genetic engineering has enabled the production of genetically truncated versions of an antibody, deprived of their constant regions (Figure 1B and 1C). These new antibody formats, rely on the assembly or randomization of CDRs of the fragment variable regions, which still possess their antigen binding properties and can be genetically fused to fusion proteins or cytotoxic payloads [78, 81, 84, 85]. These non-natural antibody fragments are of interest, since they have shown their potency in treating multiple malignancies, when genetically assembled in a specific manner [70, 78, 82, 83]. To this effect, various single chain fragment variable regions (scFvs) of about 30 kDa and consisting of the variable domain of the heavy and light chain (V_H_+V_L_) of a mAb linked by a short peptide sequence have shown their efficacies in diagnosing and treating various diseases including cancer (Figure 1C) [74, 81, 86–88].

Variable region genes of immunoglobulins can be re-assembled into multivalent antibodies with improved avidity to their target antigen. For example, diabodies of about 60 kDa can be derived from scFv fragments through engineering of their interdomain linkage; introducing a peptide with maximally five amino acids favors interchain pairing to form a dimer, while preventing intradomain linkage between V_H_ and V_L_ of the same chain [39, 86, 88, 89]. Furthermore, assembly of scFv into trimers (90 kDa) and tetramers (120 kDa) can be achieved by further reducing the length of the linker, which will ultimately increase avidity and affinity. As Fc domains are missing in these antibody formats, the cytotoxic effects cannot be driven by ADCC or CDC but could be achieved through receptor or signaling blocking and thus either obstructing the interaction between the growth factor and their target receptor or by activating downstream molecular signaling regulating cell division and cell death program [74, 81].

To conserve and expand these attributes, bispecific scFv fragments were re-engineered by joining four mAbs variable domains (V_H_ and V_L_), recognizing two different epitopes into a single chain construct. These bispecific antibodies are endowed with the capacity to recognize two different antigens, allowing cross-linkage of two different cells such as cancer cells and immune cells (e.g., T-lymphocytes, macrophages, or NK cells) [74, 86].

Altogether, these new antibody formats do have different characteristics to be exploited as therapeutic agents when compared to their mAb counterparts. These recombinant antibody formats can: (1) be genetically modified; (2) produced in different hosts of expression; and have the potential to (3) extravasate more efficiently; and (4) penetrate deeper into tumor tissue [70]. Yet, smaller size can be a limiting factor reducing their half-life in serum, due to kidney clearance filtering out molecules smaller than 60 kDa from blood and excreting them in urine [74]. Therefore, the multimerization might become a potential alternative to overcome this pre-matured excretion. Independently, protein engineering and applied chemistry is allowing to further functionalize these recombinant antibody formats by cytotoxic small molecule drugs or proteins [90, 91].

### Selected examples of a directed antibody-drug conjugation method

#### Site-specific sortase-mediated enzyme conjugation of monoclonal antibodies to cytotoxic payloads

Sortase A is a transpeptidase enzyme found within the *S. aureus* gram-positive bacteria. Sortase A induces catalysis by forming an amide bond between the threonine of the C-terminal pentapeptide (LPXTG) and the glycine at the N-terminus of the conjugation partner [56, 58]. During this transpeptidation reaction, sortase-A recognizes the C-terminus (LPXTG) sequence, cleaves the TG bond and consequently exposes the threonine to nucleophilic attack to the incoming alpha amine, which is preferably the glycine on the N-terminal of the conjugation partner *via* a thioacyl enzyme threonine intermediate [92, 93]. Recently, the antibody moiety of two ADCs (Adcetris) and trastuzumab-maytansine (Kadcyla), were used as model mAbs in the generation of sortase-conjugated ADCs [94]. These ADCs were generated through C-terminal modification of immunoglobulin heavy (IgH, anti-CD30) and light chain (IgL, anti-HER2), with sortase A enzyme recognition pentapeptide sequence (LPETG) on the targeted domain and the modification of the monomethyl auristatin E (MMAE) and maytansine payloads with pentaglycine peptide [57, 94]. Once produced, these ADCs were shown to have a higher *in vitro* killing efficacy in comparison to their non-enzymatically modified counterparts. Of note, sortase-conjugated trastuzumab-maytansine was shown to completely eradicate tumor growth in xenograft mouse models injected with HER2-overexpressing ovarian cancer cells [91]. Nevertheless, sortase A had a disadvantage since its transpeptidation reaction is limited to the C and N termini of an amino acid within the pentapeptide [94]. Based on these limitations, newer conjugation methods were developed using self-labeling proteins such as Halo, CLIP and SNAP-tag offering a unique conjugation site on the antibody, enabling the production of homogeneous ADC conjugates [56].

#### Halo, CLIP, and SNAP-tag specific conjugation methods to generate antibody-fusion proteins

Halo-Tag is an engineered version of a bacterial haloalkane dehalogenase, which is designed to rapidly and specifically react with chlorohexane-modified substrates (e.g., fluorescent dyes, affinity handles or solid surfaces), hence forming irreversible covalent bonds under physiological conditions [59, 95, 96]. Its non-human origin implies immunogenicity, in contrast to CLIP-tag and SNAP-tag which are both improved mutant versions of the human DNA repair enzyme O^6^-alkylguanine-DNA alkyltransferase (AGT) which naturally reacts with O^6^-benzylguanine derivatives [94, 96, 97]. This AGT labeling property was primarily performed through a reaction with the O^6^-benzylguanine (BG) derivative, resulting in an irreversible transfer of the BG-modified substrate to cysteine within the active site of AGT [97–99]. Subsequently, this AGT activity was improved through saturation mutagenesis experiments, which increased the AGT mutant activity by 20-fold in comparison to the wild-type [98]. Thereafter, several mutations were introduced on AGT to abrogate its DNA binding capacity, and render it resistant against inhibitors of wild-type AGT [61]. These mutations led to a reduction of the AGT size (182 residues), through the deletion of non-essential cysteine residues, which eases the folding of the mutant AGT under oxidizing conditions [100]. In summary, these mutations led to the generation of ‘suicidal enzymes’ such as CLIP-tag which can react specifically and rapidly with O^2^-benzylcytosine derivatives (BC-derivatives) and form an irreversible covalent bond between BC-ligands and cysteine residues within the CLIP-tag active site of the fusion protein. Therefore, CLIP-tag can be used as a self-labeling conjugation method for visualization of fusion proteins in living cells, as well as for enzyme-linked immunosorbent assays, western blotting, flow cytometry and immunohistochemistry [101, 102].

**Figure 2:**
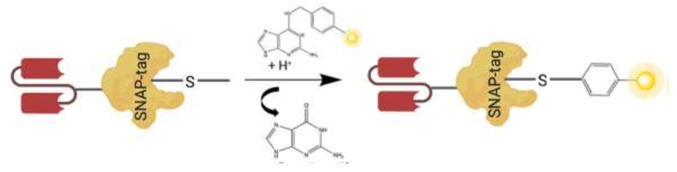
A schematic illustrating scFv-SNAP fusion protein conjugated to a BG modified substrate. Autocatalytic reaction of BG modified substrate (e.g., Photosensitizer, fluorochrome, or small molecule toxin in yellow), with the thiol group of cysteine 145 within the active site of SNAP-tag genetically fused to amino acid terminus of V_L_ chain of the scFv.

SNAP-tag is a self-labeling enzyme, resulting from an engineered version of the 20 kDa human DNA repair protein AGT that specifically and rapidly reacts with BG derivatives. SNAP-tag has a 50-fold increased reactivity with BG-modified compounds when compared to AGT, which under normal conditions functions to remove alkyl adducts from the *O*^6^ and the *O*^4^ positions of guanine and thymine to protect cells from the potent effects of alkylating agents [101, 103]. Hence, SNAP-tag performs a nucleophilic substitution reaction resulting in an irreversible, covalent coupling of BG-modified substrates, such as a fluorochrome, PS, or small molecule toxin with the thiol group of Cysteine 145, within the active site of the SNAP-tag molecule [103, 104] (Figure 2). Therefore SNAP is a simple conjugation method which ensures: (1) specificity of conjugation (reacts only with BG-modified substrates); (2) shorter conjugation reaction (30 minutes for BG-fluorochromes and 2 hours for cytotoxic payloads); (3) flexibility of expression system (bacteria, yeast, or mammalian), availability of various BG-modified substrates; (4) no reactivity with other cellular substrates; (5) no requirement for activating substrates for the conjugation reaction; and (6) a 1:1 stoichiometric reaction generating homogeneous products by only reacting with BG-molecules [88, 89, 98, 104–106].

## ANTIBODY-DRUG-CONJUGATES TARGETING EGFR

**Figure 3:**
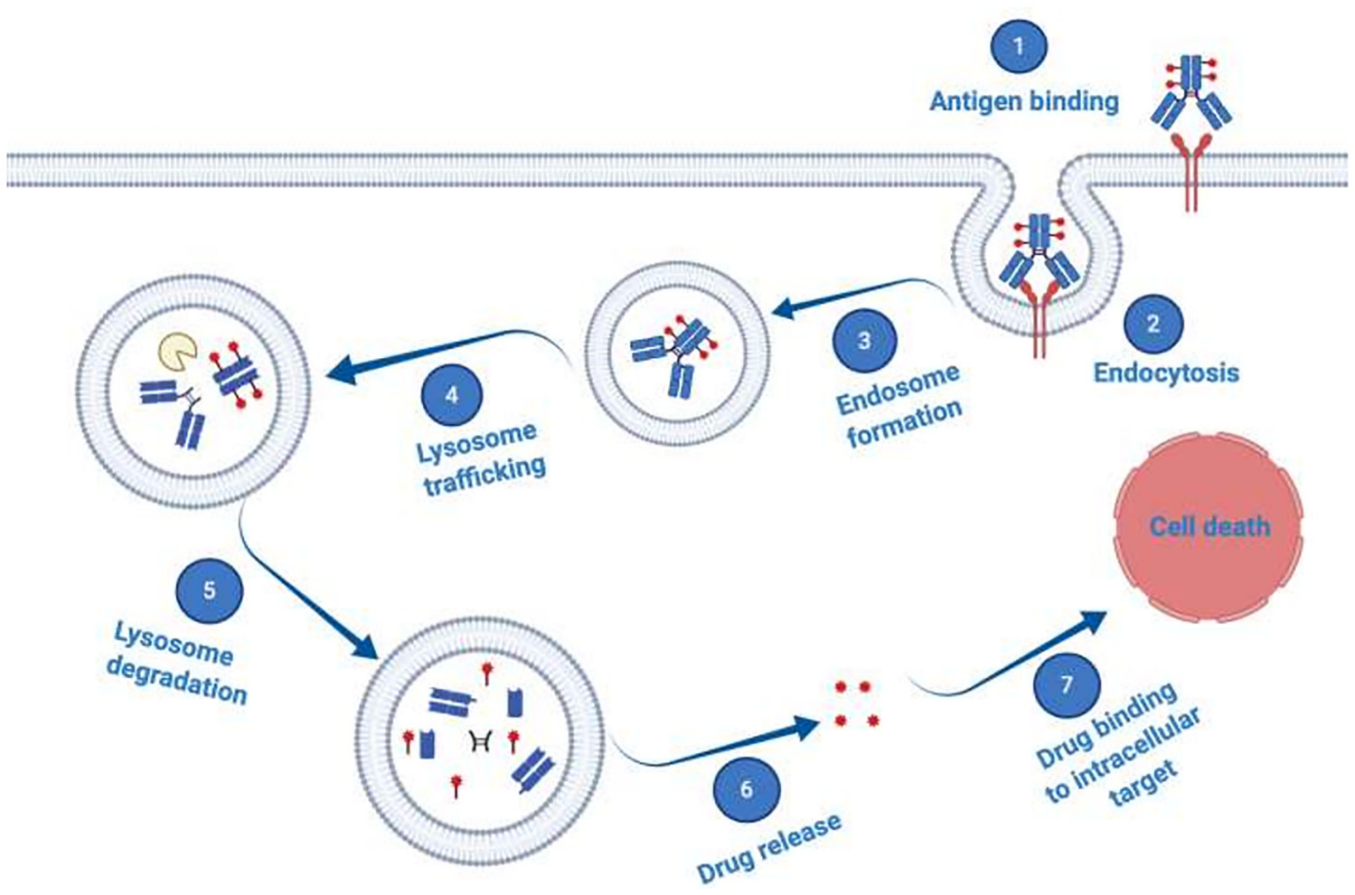
Schematic illustration of antibody–drug conjugate (ADC) mechanism of action. ADCs consist of a mAb which is attached to a synthetic cytotoxic drug through a specific chemical linker. The mAb binds to a disease-specific cognate tumor associated antigen overexpressed on target cells, is internalized *via* endocytosis and trafficked to the lysosome, where proteases degrade the ADC. Thus, the cytotoxic cargo becomes released and diffuses into the cytoplasm to reach its intracellular targets and induce cell death.

Antibody-drug-conjugates (ADCs) emerged as a promising therapeutic modality prepared from naked antibodies by chemically or enzymatically conjugating a cytotoxic payload using specific linker chemistries. Most currently, cytotoxic molecules are too toxic for systemic application, thus ADCs provide a method to harness the specificity of a mAb for targeted delivery of such highly potent cytotoxic agents to tumor cells expressing a unique cognate antigen [89, 91, 97, 104, 107, 108]. According to the generally accepted mechanism of action, binding of an ADC to such a tumor associated cell surface antigen, induces internalization of the ADC-antigen complex into the targeted cell by receptor-mediated endocytosis and subsequent trafficking of the ADC-loaded endosomes to the lysosomal compartment (Figure 3). Once in the lysosomes, the payload is released through enzymatic digestion or a pH-induced degradation of the linker, causing cytosolic release of the cytotoxic payload to efficiently induce cell death [109].

The propensity of ADCs mainly depends on the nature of the mAbs, the linker, and the cytotoxic payload, which synergistically work to exert their maximal toxicities [30, 110–112]. For instance, the chemical nature of the linker which joins the mAb to the cytotoxic payload may negatively affect ADCs’ biophysical properties, hence their potency [113, 114]. The latter was corroborated by Lewis Philips *et al.* (2008) reporting an improved therapeutic efficacy, pharmacokinetics behavior and reduced toxicity when HER2-positive breast cancer tumor xenografts were treated with a non-reducible thioether linker-based trastuzumab–maytansinoid conjugate as opposed to its counterpart that had a reducible disulfide linker [111]. Moreover, the hydrophobic nature of most potent ADCs may reduce their therapeutic efficacy as a result of mAb aggregation and precipitation [112]. To address this issue, various iterations were performed using solubilizing agents and linkers containing poly (ethylene) glycol chains of various lengths which are compatible with buffered mAbs [9]. So far, 8 ADCs including Gemtuzumab ozogamicin (Mylotarg), Brentuximab vedotin (Adcetris), Trastuzumab emtansine (Kadcyla/T-DM1), Inotuzumab ozogamicin (Besponsa), Polatuzumab vedotin-piiq (Polivy), Enfortumab vedotin (Padcev), Trastuzumab deruxtecan (Enhertu), and Sacituzumab govitecan (Trodelvy) have received FDA approval for cancer treatment [115]. Additionally, AMG-595 and depatuxizumab mafodotin (Depatux-m), both targeting EGFR VIII overexpressing glioblastoma multiform (GBM), were developed and improved therapeutic efficacy when treating GBM in both preclinical and clinical studies, through specific intracellular delivery of cytotoxic anti-microtubule agents such as maytansinoid or monomethyl auristatin F (MMAF) [116]. Conventionally, ADCs are generated through chemical conjugation (alkylation or acetylation) of lysine, or reduced inter-chain disulphide residues of mAbs to cytotoxic payloads [117–122]. In 2014, an orphan drug status (assigned to a medicine intended for use in rare diseases) was given by the FDA to Depatux-m also known as ABT-806 [94, 111, 123]. Depatux-m consists of an IgG1 humanized antibody, conjugated to MMAF using a non-cleavable linker maleimidocaproyl [120–122, 124]. This ADC targets a unique EGFR epitope variant (EGFR VIII), which is genetically deprived of exons 2 to 7 and commonly found in GBM, the most common form of malignant brain cancer [120–122, 124, 125]. EGFR VIII defines a unique epitope lacking an ectodomain and is associated with GBM poor prognosis (a median survival of about 16 months upon diagnosis), caused by constitutive activation of its intracellular tyrosine function [120–122, 124, 126]. Under normal physiological conditions, EGFR VIII is not accessible and not expressed on normal cells [120, 121, 123]. This makes it an ideal biomarker, limiting the undesirable side effects associated with ADCs. The results of several pharmacological studies revealed that a drug-to-antibody ratio (DAR) of 4 was required for depatux-m to significantly reduce tumor growth both in preclinical and clinical studies [121, 122, 124]. Interestingly, Depatux-m was shown to bind EGFR VIII with higher affinity than cetuximab and to synergistically increase therapeutic efficacy when combined with standard care therapies (e.g., Cisplatin, 5-FU and temozolomide) when treating GBM and HNSCC [117–122, 127]. Depatux-m has passed phase I clinical trial and its efficacy is presently being assessed in phase II/III clinical trials on EGFR overexpressing GBM patients (NCT02343406, NCT02573324). These results spurred the development of other EGFR specific ADCs such as AMG-595, consisting of a fully human EGFR IgG1 mAb linked to the antimitotic agent maytansinoid DM1 *via* a non-cleavable maleimidomethyl cyclohexane-1-carboxylate (MCC) linker [121]. According to Hamblett *et al*. (2015), AMG-595 exclusively binds to and kills EGFR VIII expressing GBM both in *in vitro* and in preclinical orthotopic mouse xenograft models using a DAR of about 3.5 [128]. Recently, Rosenthal *et al.* (2019), have shown during a phase I clinical trial that AMG-595 was safe to treat GBM patients overexpressing EGFR VIII [128]. According to this phase I trial, 47% and 6% of patients respectively had a stable disease (17 out of 32) and a partial response (2 out of 32) correlating with a dose-dependent increase of conjugated products in plasma and a very low level of unconjugated antibody and cytotoxin [126]. Regardless of these therapeutic successes, multiple factors such as the type of linker must be taken into consideration to predict the clinical efficacy of ADCs, as unstable linkers and ADC recycling by the neonatal Fc receptor (FcRn) upon internalization, have the capacity to prolong their systemic circulation which can potentially cause side effects [126]. Other factors including DAR have shown to negatively impact the pharmacokinetic behavior of ADCs, and the therapeutic outcome due to generation of heterogeneous ADC products, which can aggregate and precipitate in virtue of the payload hydrophobic properties [30, 125, 129, 130]. This has been mitigated by the use of solubilizing agents [9]. Interestingly, a DAR of 4 was found to reduce the presence of unconjugated antibody and maintain the half-life circulation of ADCs [131–134]. On the other hand, a DAR of 8 showed to cause ADC deterioration, increase premature clearance from bloodstream and aggregation capacity, which induces an immunogenic reaction as a result of the hydrophobic nature of the payloads, while reducing their stability under stress conditions [31]. Contradicting results using similar DAR (8) were recently published by Iwata *et al*. (2018 and 2019), showing the antitumor efficacy of trastuzumab deruxtecan (Humanized anti-hHER2 conjugated with the topoisomerase I inhibitor exatecan derivative DS-8201) using a mouse model of colon and breast cancers overexpressing HER2 receptor [135, 136]. DS-8201 was exerting its therapeutic efficacy by specifically killing HER2 expressing tumors and increasing tumor infiltrating dendritic cells, CD4^+^ and CD8^+^ T-cells *in vivo* [137, 138]. Of late, DS-8201 has gained FDA approval (December 2019), and has clinically shown its efficacy in treating various malignancies including: breast, gastric, gastro-esophageal, colorectal, salivary, and non-small cell lung cancers [130, 137–140]. The success of DS8201, was based on improved therapeutic properties including: good homogeneity of high DAR, tumor selective cleavable peptide linker, with increased linker-payload serum stability and short half-life of the toxic-warhead [141]. Furthermore, the nature of the linker, which covalently connects the cytotoxic payloads to the mAb is crucial as it may significantly impact ADC activities. The ideal linker should be stable enough to maintain the cytotoxic payloads attached to the mAb and only release it once internalized within cancer cells [30, 110, 123, 142]. Inevitably, one needs to critically evaluate the linker design, prior to the engineering of an ADC. Two classes of ADC linkers can be distinguished based on their capacity to be cleaved or not, once internalized within the target cells [110, 143]. Among the cleavable linkers, multiples subtypes such as acid labile linkers (e.g., hydrazine linkers), were used to produce the FDA approved Gemtuzumab ozogamicin. These hydrazine linkers are pH sensitive and usually dissociate from mAbs through hydrolysis in lysosome-like microenvironments—very acidic—or hypoxic tumor regions [56, 93, 144]. Other forms of cleavable linkers include valine-citrulline dipeptides and disulphide linkers, which respectively rely on enzymatic cleavage (e.g., cathepsin B, cysteine protease) under acidic lysosomal conditions and high level of reduced glutathione [31, 145]. Conversely to cleavable linkers, non-cleavable linkers are inherently stable in plasma with reduced side effects, which favor repeated treatment cycles [31, 110]. It then ensues that the type of chemical conjugation of the payloads to ADCs are very critical, as they significantly influence ADC stability, clinical efficacy, DAR and pharmacokinetic behavior [137, 146]. Examples of non-cleavable linkers include lysine or cysteine amino acid conjugations, which tend to generate different DARs or necessitate partial cysteine reduction [31, 147]. Lately, efforts to improve ADCs homogeneity through site-specific conjugation of mAbs to toxic payloads, have been performed using SNAP-tag technology.

### EGFR-targeting SNAP-tag based antibody fusion proteins

Lately, SNAP-tag was used to generate several recombinant antibody-based fusion proteins for photoimmunotheranostic (PIT) and ADC-based treatments in melanoma, ovarian and breast cancers [31, 39, 88, 89, 107, 111, 148]. EGFR expressing tumors were selectively killed by conjugating specific scFv-SNAP fusion proteins to near infrared PSs (such as IR700) or auristatin F (MMAF/AURIF) [39, 83, 84, 106, 139]. Binding and internalization of the anti-EGFR immunoconjugate 425(scFv)-SNAP-AURIF was confirmed on EGFR-expressing target cells confirming that conjugation to MMAF (or AURIF) did not influence the binding activity of the fusion protein as expected, since the active site of SNAP-tag is structurally opposed to the paratope of the scFv [39, 88, 89, 107, 149]. Additionally, while unconjugated BG-AURIF was toxic to all cell lines, 425(scFv)-SNAP-AURIF did not affect the viability of EGFR-negative A2058 control cells [149]. This implies that the specificity and functionality of the antibody moiety is still retained. In contrast to auristatins which are known to show cytotoxicity in the lower nanomolar range (~1 nM) [149], the authors were able to show comparable cytotoxicity, ranging from 3–21 nM (based on the cell line), indicating that AURIF retained its anti-tumor activity even after being BG modified [150]. Furthermore, the stability of ADCs in circulation is critical to limit side-effects caused by systemic application and 425(scFv)-SNAP-AURIF was able to maintain at least 50% of its initial cytotoxicity after 48 hours incubation in serum [149]. Selective binding to EGFR-positive breast cancer and other overexpressing tumor cell lines described for 425(scFv)-SNAP-AURIF and panitumumab-derived 1171(scFv)-SNAP-AURIF, was also confirmed on EGFR-positive breast cancer biopsies [149]. A DAR of 1 in these types of recombinant SNAP-tag based ADCs (Table 1) may be explored to further increase the efficacy of ADCs by novel synthetic chemistries without abrogating binding activity [107]. In conclusion, SNAP-tag allows the stable and efficient linkage of AURIF to recombinant antibody fragments, thus offering a promising avenue to improve the development of personalized medicines [149]. For these reasons, the use of SNAP-tag fusion proteins as a targeted therapeutic approach, might become a pertinent choice in combating chemotherapy-resistant cancers.

**Table 1:** Cytotoxic activity of EGFR-specific recombinant antibody-drug conjugates

EGFR-specific immunotherapy	Construct name	Disease model	IC_50_value	References
***Recombinant Antibody-Drug Conjugates***
	425(scFv)-SNAP-AURIF	Epidermoid carcinoma	8 nM	[107, 149]
		Triple-negative breast cancer	2.6 nM–4 nM	
		Rhabdomyosarcoma	8 nM	
	1711(scFv)-SNAP-AURIF	Epidermoid carcinoma	12 nM	[107]
		Triple-negative breast cancer	4 nM	
		Rhabdomyosarcoma	4 nM	
	αHER2(scFv)-SNAP-AURIF	Breast cancer	0.6 nM	[149]

## EGFR-TARGETING ANTIBODY PHOTOIMMUNOCONJUGATES

### Near-infrared photoimmunotherapy

**Figure 4:**
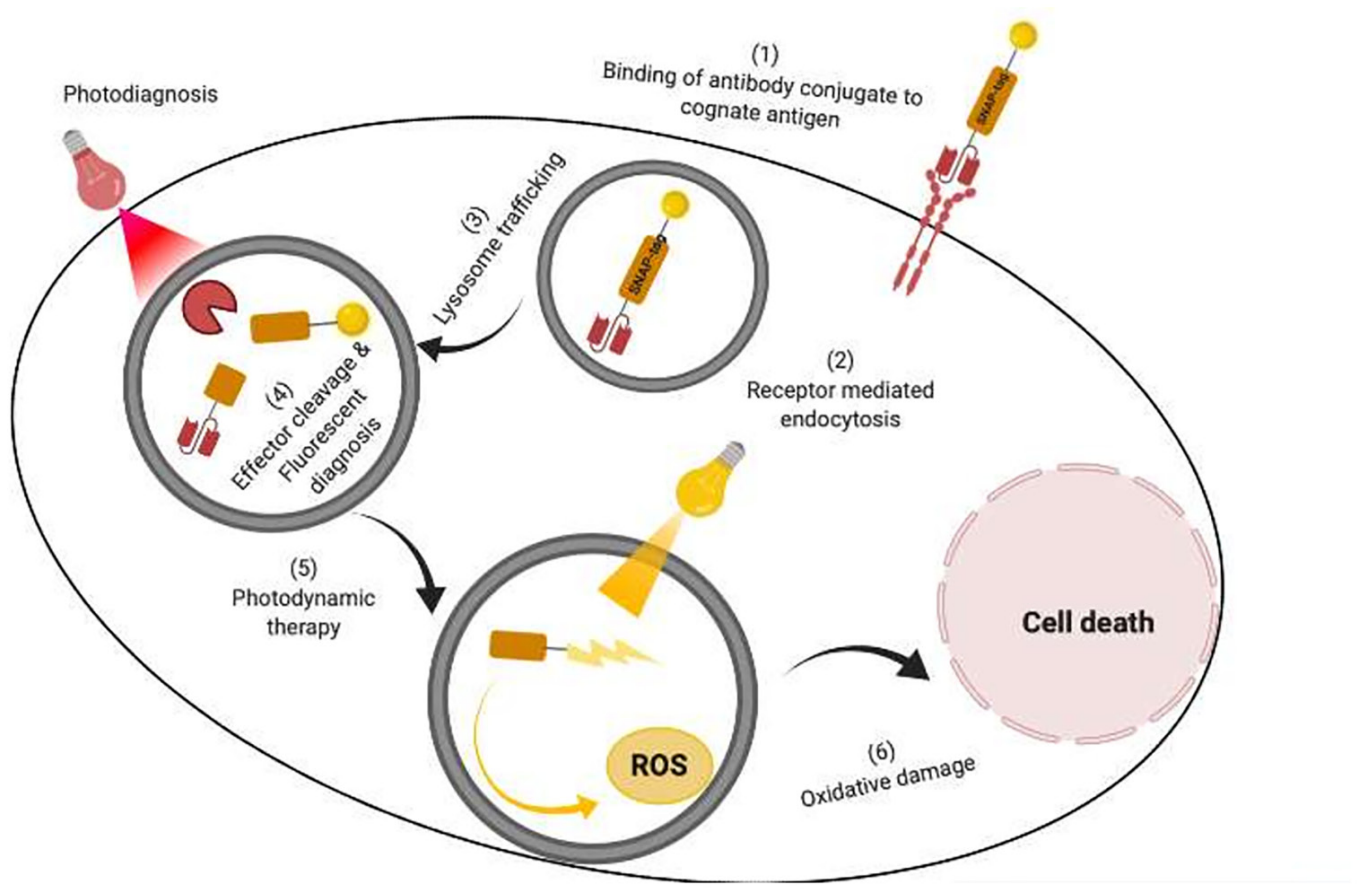
An illustration of targeted delivery of photoimmunotheranostic agent to specifically detect and kill cancer cells. (**1**) The SNAP-tag antibody photoimmunoconjugate first binds to the cognate receptor expressed on targeted cancer cells. (**2** and **3**). Thereafter the APC is internalized through receptor mediated endocytosis into the cell and trafficked to the lysosome. (**4**) The APC is subsequently exposed to a specific wavelength of light which enables fluorescent based detection of the targeted cell. (**5**) Irradiation of the APC with a different therapeutic wavelength in the presence of molecular oxygen causes reactive oxygen species (ROS) production which oxidatively damage the cell and induce cell death.

Near-infrared photoimmunotherapy (NIR-PIT) can be defined as the targeted version of photodynamic therapy (PDT), an FDA-approved anti-cancer modality using a light-activated compound known as a PS, to produce death-inducing amounts of reactive oxygen species (ROS), causing tumor destruction through apoptosis, necrosis, vasculature damage, and initiation of acute local and systemic inflammation (Figure 4) [107]. As opposed to PDT, NIR-PIT utilizes the specificity of a tumor-specific mAb conjugated to a PS (e.g., phthalocyanine dye IR700) to induce phototoxicity after NIR light exposure (e.g., 690 nm) [9, 144, 151, 152]. Recently, multiple *in vitro* studies have shown the specificity and efficacy of NIR-PIT in killing targeted cancer cells using sub-nanomolar concentration ranges of the PS which were non-toxic to healthy neighboring cells (Table 2) [42, 88]. Similarly, numerous preclinical human xenograft models of breast, melanoma, glioblastoma, ovarian, and pancreatic cancers have shown the combined potent therapeutic and diagnostic effects of NIR-PIT in reducing tumor growth [7, 10, 39, 41, 86, 144–146]. For instance, Burley *et al.* (2018), showed that reduction in cellular proliferation and GBM tumor growth could be achieved using EGFR VIII-targeted NIR-PIT [9, 12, 39, 41, 91, 153–155]. This result was conforming with Ito *et al*. 2016 study, showing the synergistic potential of trastuzumab and pertuzumab-targeted NIR-PIT (using IR700) in reducing HER2-overexpressing breast and gastric tumors [155]. Comparable effects were obtained by Nakajima *et al.* (2013), when combining panitumumab-IR700 with basiliximab-IR700 (interleukin-2 receptor alpha CD45) to targeted cancer cells expressing these receptors [154]. Also, Sato *et al.* (2014) showed that panitumumab-IR700 was more efficient than cetuximab-IR700 in killing EGFR overexpressing tumors [41]. This differential *in vitro* therapeutic efficacy was associated with faster hepatic catabolism and poor cetuximab-IR700 tumor penetration when compared to panitumumab-IR700 [12]. Besides, Saxena *et al.* (2015), revealed that post-operative NIR-PIT could significantly reduce both local and metastatic pancreatic tumor recurrence, when compared to bright light surgery (BLS) which displayed bigger tumor volume (115.2 mm^2^) than its counterparts (2.14 mm^2^) [12]. The preclinical success of these studies paved the way for the first cetuximab-IR700 human clinical trial which has reached phase III and is presently being tested for the treatment of advanced head and neck cancer patients with recurrent disease (NCT03769506) [40].

**Table 2:** Cytotoxic activity of EGFR-specific antibody photoconjugates

EGFR-specific immunotherapy	Construct name	Disease model	IC_50_value	References
***Antibody photoconjugates***	scFv-425-SNAP-IR700	Epidermoid carcinoma	32 nM	[89]
	scFv-425-SNAP-IR700	Melanoma skin cancer	45 and 55 nM	[89]
	scFv-425-SNAP-IR700	Triple-negative breast cancer	26–69 nM	[88]
	scFv-45-SNAP-IR700	Isolated ascites	40–90 nM	[39]
		Ovarian cancer	45–66 nM	[39]
	Panitumumab-IR700	Breast cancer	67.57 nM^*^	[156]
	Pertuzumab-IR700	Breast & gastric cancer	67.57 nM^*^	[154]
	Trastuzumab-IR700	Breast & gastric cancer	67.57 nM^*^	[154, 157, 158, 160]
	Z-EGFR03115-IR700DX	Glioblastoma cancer	0.5–0.1 μM	[155]

^*^Molar IC_50_ values calculated from the molecular weight of the fusion protein and the reported IC_50_ values (g/ml).

### Photoimmunotheranostic treatment

Photoimmunotheranostic treatment is a new treatment strategy combining the diagnostic and tumor shrinkage properties of antibody photoimmunoconjugates (APCs), which specifically accumulate into targeted tumors and induce their selective destruction upon irradiation with a specific light source (Figure 4) [9, 156–160]. This novel cancer treatment approach offers promising opportunities in improving cancer detection and monitoring post-treatment responses [161]. Taking this into consideration and using SNAP-tag technology, von Felbert *et al*. were able to specifically visualize and kill skin cancer cells *in vitro* using a panitumumab-derived SNAP-IR700 showing IC_50_ values of 32–55 nM (Table 2) [11]. These results were supported by reports from Amoury *et al*. (2016) and Bauerschlag *et al*. (2016) demonstrating the use of SNAP-tag conjugates for the detection of tumor sections in ovarian and breast cancer tumor biopsies (overexpressing CSPG4, EGFR, and EpCAM) using fluorescence immunohistochemistry and the induction of targeted killing with IC_50_ values of 45–90 μM and 62–165 μM, respectively (Table 2) [89]. Of note, triple-negative breast cancer (TNBC) is the most aggressive form of breast cancer comparably resistant to conventional therapies and cannot benefit from hormone therapies due to absence of corresponding receptors [88]. TNBC therapeutic resistance has partly been associated to a therapeutic resistant subpopulation overexpressing CD44. Hence, using theranostic treatment, Jin *et al*. (2016) were able to specifically detect and destroy CD44-expressing tumors in a human xenograft tumor model [144]. This targeted regimen is of particular clinical relevance, since it can reduce post-operative residual TNBC tumors using image-guided surgery or specifically treat therapy resistant primary tumors [153]. Interestingly, a study led by Ogata *et al*. (2017) revealed the superiority of repeated NIR-PIT (2 or 3 times on the same day) in significantly reducing tumor growth and prolonging overall survival compared to single treatment [153]. Although only demonstrated in animal models, this therapeutic regimen should be more effective and more economically viable for patients, who would only require a single dose of APC, followed by multiple irradiation doses to achieve greater tumor responses [11]. Currently the preferred regimen consists of injecting APCs 24 hours before the first irradiation. Thus, this implies that a second or third irradiation would activate circulating APCs, which did not previously accumulate within the tumor [11]. With this in mind, Harmatys *et al*. developed a long circulating pyropheophorbide (LC-Pyro) immunoconjugate (conjugate to prostate specific membrane antigen) with a trifunctionality capable of screening tumors using fluorescent imaging and positron-emission tomography (PET) [162]. Using this LC-pyro immunoconjugate, this group was able to diagnose tumors in orthotopic, subcutaneous and metastatic murine animal models [162]. Upon light activation, the LC-pyro immunoconjugate was able to significantly increase overall survival (over 40 days) when compared to the unirradiated control (24 days) [162].

## EGFR-SPECIFIC RECOMBINANT IMMUNOTOXINS

### Anti-EGFR immunotoxins in cancer therapy

The development of immunotoxins (ITs) was the logical consequence of an alternative strategy to antibody-driven chemotherapeutics, based on the speculation that replacing inefficient synthetic warheads by protein toxins with enzymatic activity should theoretically allow to kill cells with only a few effector molecules released within the target cell [162]. ITs are potent molecules consisting of a protein toxin linked to a binding ligand such as an antibody or a growth factor [163]. Whereas the first generation of ITs was based on chemical conjugation, successive generations were primarily recombinant versions with higher specificity, reduced toxicity and improved tumor penetration, while also ensuring large-scale protein production at high purity and quality [164, 165]. As stated by Chandramohan *et al*. (2013), the therapeutic success of a tumor-targeting agent is dependent on 2 critical factors: (1) efficient delivery to the tumor site at adequate concentration; and (2) uniform distribution throughout the neoplastic lesion [166]. ITs satisfy these criteria through their ability to bind with high specificity to surface antigens, causing their internalization and killing of the tumor cell by catalytic inhibition of protein synthesis within the cell cytosol [167]. Early developers of ITs were able to harness the activity of various plant and bacterial toxins. Toxins such as diphtheria toxin (DT), ricin A or Pseudomonas exotoxin A (also known as ETA or PE) are endowed with their own translocation domains and other components that can facilitate endosomal escape – a major rate-limiting step in the delivery of therapeutic macromolecules to the cytoplasm of cells [164]. With this peculiar characteristic, such toxins display higher toxicity than chemical agents; a single toxin molecule is enough to kill a cell while 10000 to 100000 molecules of toxic chemicals are required to produce the same effect [168–171].

In comparison to mAbs and ADCs, recombinant ITs display improved tumor penetration capability and greater anti-tumor efficacy in preclinical cancer models [172]. Furthermore, their unique features, including high specificity, extraordinary potency and lack of drug resistance, provided a rationale for the development of various EGFR-targeting ITs [173]. In order to target tumor cells *via* a surface molecule such as EGFR, the anti-tumor fusion toxin must: (1) recognize and bind with high affinity; (2) exhibit high selectivity for EGFR-overexpressing tumor cells to minimize unwanted side-effects against normal tissues eventually expressing low levels of target antigen; and (3) have a catalytically active cytotoxic domain effectively inducing apoptosis at low concentrations [173]. Therefore, to achieve these requirements, EGFR has been under rigorous scrutiny, resulting in the generation of novel anti-EGFR ITs based on different growth factors, mAb templates and plant/bacterial toxins. Some examples include anti-EGFR (scFv)-rGel (anti-EGFR single-chain antibody fragment-Gelonin), DAB_389_EGF (DT toxin-EGF fusion protein), Sap3-EGF (Saponin toxin-EGF fusion protein), anti-P170^EGFR^-RTA (anti-EGFR mAb-Ricin A Chain) and TGFα-PE_40_ (Transforming Growth Factor type alpha-PE toxin with domain I deleted) [174]. While these toxins and growth factors have shown promising activity for a number of EGFR-driven malignancies, recombinant Fv variants conjugated to PE (or deimmunized PE variants), have been most commonly used to enhance cytotoxic activity, reduce immunogenicity, improve penetration into solid tumors and reduce the clearance time through the kidneys and liver [175–179].

Indeed, PE is a widely studied bacterial toxin consisting of a 613-residue, arranged in 3 separate regions: domain I is responsible for cell recognition (domain Ia as the cell-binding domain and domain Ib of unknown function), domain II for translocation into the cytosol and lastly, domain III which irreversibly inhibits protein synthesis by adenosine diphosphate (ADP)-ribosylation of human elongation factor 2 (EF-2) [172, 174, 180]. For recombinant IT development, researchers replaced the original cell-binding domain of PE by specific ligands or scFvs to allow specific targeting of tumor cells and efficient cell killing activity following internalization [163, 181, 182]. Moreover, various forms of PE (including PE40, PE38 and PE25) have been investigated to obviate impediments resulting from the immunogenicity of the toxin moiety [183]. More explicitly, PE40 (40 kDa) was generated by the elimination of domain Ia and PE38, by the removal of a large part of domain Ib, without compromising the cytotoxicity and ADP-ribosylation activity [172]. Similarly, to further reduce the immunogenicity and side-effects encountered with PE38, the smallest version of PE was engineered (known as PE24), which lacks domain II of PE, with exception of a 11 amino acid length furin cleavage site [163, 184] and which might show differences in cytotoxicity dependent on intracellular routing. The furin cleavage site plays a critical role in the intracellular processing of the toxin [185]. Additionally, the natural C-terminus REDLK sequence of PE was modified to KDEL to increase intracellular retention and cell-killing activity [186, 187].

Strategies based on anti-EGFR recombinant ITs bearing PE variants as lethal warheads, have thus shown promising results in several studies [184] (Table 3). Because of EGFR’s role in the malignant process, elevated expression and accessibility on the tumor cell surface [163, 167, 174, 182, 188–190], EGFR-specific ITs represent highly potent immunotherapeutic agents across a wide range of diseases, including glioblastoma, breast, prostate and pancreatic cancer. Most strikingly, Niesen *et al*. (2015), have described the engineering and functional characterization of 2 novel recombinant ITs (scFv1711-ETA’ and scFv2112-ETA’) based on panitumumab and cetuximab [174]. These ITs showed significant pro-apoptotic and anti-proliferative effects towards target cells, with IC_50_ values ranging from 4 to 460 picomolar (pM), depending on the EGFR expression level (Table 3) [188]. In comparison, the IC_50_ values of the internal reference 425(scFv)-ETA’ were similar or slightly better than the new ITs, with lower IC_50_ values observed against cell lines expressing the highest level of EGFR [188]. These results were in line with a previous report demonstrating a clear correlation between EGFR expression and the EGFR-specific IT [182, 188, 189]. In addition to the level of target receptor, other cell-type specific factors such as the rate of receptor turnover could strongly influence IT sensitivity [191].

**Table 3:** Cytotoxic activity of EGFR-specific immunotoxins and human cytolytic fusion proteins

EGFR-specific immunotherapy	Construct name	Disease model	IC_50_value	References
***Recombinant Immunotoxins***	D2C7-(scdsFv)-PE38KDEL	Glioblastoma	2.9–40.32 pM^*^	[167]
	scFv (225)-ETA	Squamous cell carcinoma	< 14.29–271.43 pM^*^	[174, 190]
	scFv (14E1)-ETA	Squamous cell carcinoma	27.97–111.89 pM^*^	[174, 190]
	425(scFv)-ETA’	Squamous cell carcinoma	2 pM	[182, 188, 189]
		Breast cancer	4 pM	
		Prostate cancer	35 pM	
		Pancreatic cancer	80 pM	
		Rhabdomyosarcoma	598 pM	
	Humanized anti-EGFR (huscFv)-PE25KDEL	Epidermoid carcinoma	9.43 nM^*^	[163]
	scFv1711-ETA’	Squamous cell carcinoma	18 pM	[188]
		Breast cancer	32 pM	
		Prostate cancer	192 pM	
		Pancreatic cancer	260 pM	
		Rhabdomyosarcoma	240 pM	
	scFv2112-ETA’	Squamous cell carcinoma	4 pM	[188]
		Breast cancer	11 pM	
		Prostate cancer	55 pM	
		Pancreatic cancer	290 pM	
		Rhabdomyosarcoma	460 pM	
***Human Cytolytic Fusion Proteins***	αEGFR (scFv)-MAP tau	Pancreatic carcinoma	1000 nM	[211]
		Prostate cancer	2500–2800 nM	
	scFv1711-GrBR201K	Epidermoid carcinoma	133.3 nM	[35]
		Rhabdomyosarcoma	21.2 nM	
	αEGFR (scFv)-Angiogenin	Epidermoid carcinoma	12.5–45 nM	[212]

^*^Molar IC_50_ values calculated from the molecular weight of the fusion protein and the reported IC_50_ values (g/ml).

Researchers have found that the overexpression of EGFR, is often accompanied by an increased production of the EGF receptor ligand TGF-α, which results in receptor activation by autocrine stimulation and ultimately fosters malignant transformation [192]. In contrast to the EGFR-specific ITs, mAbs do not have the ability to kill tumor cells directly, but instead, they inhibit ligand binding, block signal transduction and inhibit EGFR gene expression [193, 194]. Schmidt *et al.* were interested in developing the EGFR-directed ITs, scFv (225)-ETA and scFv (14E1)-ETA, which (like their parental mAb) are able to competitively inhibit the binding of EGF and TGF-α to the EGF receptor, thereby blocking receptor activation [195].

Nonetheless, despite their high potency and affectivity, recombinant ITs face several disadvantages which limit their overall anti-tumor efficacy in clinical applications. The repeated use of high concentrations of these toxins gave rise to side-effects such as liver injury and vascular leak syndrome [174]. Moreover, some of the current ITs have low binding affinity with EGFR due to their monovalency and their effectiveness is further hindered by the cross-reactivity with EGFR on normal tissues [196, 197]. To address this problem, Meng and colleagues proposed the use of a bivalent recombinant anti-EGFR IT (DT390-BiscFv806) which showed promising activity against various cancers [173]. While several PE-based ITs have demonstrated potential in clinical and preclinical studies [173], the non-human effector component provoked an immune response, which leads to dose limitations.

To this end, various humanization approaches have been proposed: treating patients with immunosuppressive drugs, chemically modifying proteins *via* PEGylation [198–200], removal of human B-cell and T-cell epitopes from plant/bacterial toxins by site directed mutagenesis [201], or substituting bacterial/plant toxin moieties with toxins of human origin (to give rise to fully human cytolytic fusion proteins or hCFPs) [202, 203].

### Reducing immunogenicity: immune modulating drugs and the de-immunization of Pseudomonas Exotoxin A

The therapeutic efficacy of recombinant ITs in clinical trials is considerably hampered by the formation of neutralizing antibodies [32, 188]. This phenomenon often results in immune-related adverse events (such as allergic skin reactions and anaphylaxis) which further jeopardize the possibility of favourable treatment outcomes [204]. Therefore, several strategies have been put forward to mitigate the impact of immunogenicity on the therapeutic success of these agents. For instance, ITs are being used in combination with immune modulating drugs; in year 2004, 5 patients were pre-treated with rituximab to eliminate their B cells prior to LMB-1 (a mAb targeting Lewis Y-related B3 epitope with PE38) administration. However, all patients developed neutralizing antibodies by day 21 of drug administration, indicating that the elimination of B cells is not adequate to counteract an immune response [200, 205]. To this end, Pentostatin was used to abrogate the activity of T-cells, along with Cytoxan (to eliminate B cells) in the treatment of mesothelioma patients with SS1P (an anti-mesothelin PE-based IT) [206]. Furthermore, with substantial progress in protein deimmunization by the Pastan group, the first “de-immunized” PE-based mesothelin-targeting IT was engineered, most commonly known as LMB-100, consisting of a humanized Fab fused to LO10 (PE toxin with 7 major B cell epitopes silenced) [191]. While T-cell de-immunization efforts have not yet been assessed in the clinical setting, the B cell de-immunized IT, LMB-100 has recently been tested in 2 clinical trials. From the results generated (https://clinicaltrials.gov/: NCT03436732, NCT03644550), it was concluded that while using a humanized antibody and the silencing of B-cell epitopes is promising, it is not sufficient to completely abolish an immune response against recombinant ITs. Consequently, the arguments above warrant the need to intensify research for alternative strategies to alleviate the impacts of immunogenicity. This would also be a salient point to consider in the development of next-generation EGFR-specific recombinant ITs.

## HUMAN CYTOLYTIC FUSION PROTEINS TARGETING EGFR

The 4th generation of ITs, also known as targeted human cytolytic fusion proteins (hCFPs) represent a combination of fully human sequences for the antibody, as well as the cytotoxic module [207]. To this end, a portfolio of very potent endogenous proteins of human origin have been identified as potent candidates for the production of less or non-immunogenic ITs. These include granzyme B (GrB), immunoRNAses (such as Angiogenin [Ang]), death-associated protein kinase and the microtubule-associated protein tau (MAP tau), amongst others [32, 33, 172, 208]. For the selective elimination of tumor cells *via* apoptosis, hCFPs must be able to bind to the target antigen and be effectively internalized, followed by endosomal escape and release of the cytotoxic cargo into the cytosol of the cell. The higher IC_50_ values observed with hCFPs as compared to PE-based ITs (Table 3), reveal that there is a need for translocation promoting structures in the natural human enzymes. In order to improve the cytotoxic activity, endosomolytic compounds, such as chloroquine or wortmannin, could be used [32, 209]. Nonetheless, recent studies point to the use of adapter sequences that facilitate vesicular escape of the effector molecule into the cytosol of the tumor cell [32, 210]. Here, we review the past and current research conducted in the context of EGFR-targeted hCFPs bearing GrB, Ang, or MAP tau [32, 33, 210, 211].

### Granzyme B

Granzyme B (GrB) is a cytolytic serine protease found in granules of innate immune effector cells (natural killer and cytotoxic T-lymphocyte cells), which functions to protect the body against viral infections and malignant cells [35, 211–214]. Due to its cytotoxic nature, GrB exists as a zymogen with an N-terminal signal sequence which can be processed in the endoplasmic reticulum and post-translationally modified with mannose-6-phosphate, priming it for packaging with serglycin and perforin complex into secretory vesicles [31]. During cytolytic destruction of targeted cells by cytotoxic T-lymphocytes (CTLs), cytotoxic granules are released at the intercellular spaces called immunological synapses [32, 215]. Thereafter, perforin is released to polymerize and create transmembrane pores on targeted cell membrane, to ease GrB access to molecular cytosolic targets [204]. Alternatively, GrB can enter targeted cell cytosol through the endosomolytic perforin activity, following mannose-6-phosphate receptor mediated endocytosis [216, 217]. Once in the cytosol, GrB can catalytically cleave and produce truncated versions of pro-apoptotic proteins of the BcL-2 family such as BH3 interacting domain death agonist (BID), which eventually translocates to the mitochondria, causing cytochrome c release and activating downstream apoptotic signals inducing DNA damage, hence cell death [33]. The human origin of GrB makes it an ideal candidate as an effector molecule for the generation of recombinant hCFPs capable of circumventing the adverse effects (e.g., immunogenicity and side effects) usually associated with plant and bacterial toxins [32, 33]. In this regard, Liu *et al.* (2003), developed a GrB which was genetically fused to a single chain anti-melanoma antibody fragment (anti-gp240) that could specifically induce apoptosis in targeted cells 8 hours post-treatment with IC_50_ values of 20 nM [32, 36, 217, 218]. Corroborating results from Dälken *et al*. and Oberoi *et al*. supported the specificity and therapeutic efficacy of TGFα-GrB hCFPs in killing EGFR overexpressing cancer cells using pico to nanomolar IC_50_ concentrations in the presence of endosomolytic chloroquine reagent [217, 219]. However, GrB hCFP targeted therapy is usually impaired by the presence of the endogenous inhibitor serine protease serpin B9, naturally protecting CTLs from granules-loaded GrB [36, 215]. To exert its inhibitory effect, serpin B9 irreversibly binds to GrB in a 1:1 stoichiometry which is stabilized by various chemical interactions including hydrogen and hydrophobic bonds [36, 220]. Using computational modelling and recombinant antibody technology, both Niesen and Amoury *et al*. (2016) developed an inhibitory resistant version of GrB (201K), genetically fused either to panitumumab (scFv1711) or EpCAM scFv fragment [35, 221]. This new GrB version (Gb201K-αEpCAM) could kill αEpCAM overexpressing tumor cells using nanomolar IC_50_ concentrations which were three fold lower than conventional IT conjugates (αEpCAM (scFv)-ETA counterparts) [34, 200]. Besides this, Gb201K-αEpCAM did not cause any side effects, which offers better therapeutic tolerability, allowing repeated treatment schedules with higher doses, which are normally limited in highly immunogenic bacterial ITs treatment as a result of antibody-neutralizing immune responses [35, 213].

### Angiogenin

Angiogenin (Ang) or ribonuclease 5 (RNase5), is a 14 kDa stress-activated enzyme belonging to the pancreatic ribonuclease (RNase) superfamily, which possesses angiogenic and ribonucleolytic activities [213, 222]. Using its nuclear ribonuclease activity, Ang has shown to primarily function to regulate angiogenesis and positively influence the activation of molecular pathways driving cancer’s metastatic, invasive and migratory potential [33, 36, 211]. Also, Ang has shown the ability to translocate in the cell cytosol in response to oxidative stress and induce apoptosis by abolishing protein synthesis through tRNA, 5S, 18S, and 28S rRNA hydrolysis [34, 36, 223, 224]. Unfortunately, Ang therapeutic efficacy has been hampered by the antagonistic effect of the endogenous human placental ribonuclease inhibitor 1 (RNH1), which acts to prevent self-tissue damage [34, 212, 225, 226]. To bypass this obstacle, Cremer *et al.* (2015) and Gresch *et al*. (2018), engineered multiple Ang mutant versions, which have decreased affinity for their RNH1 [34, 223]. According to these studies, the Ang mutants were associated with increased cytotoxicity towards CD64 (activated macrophages) and CD89 positive cells (acute myeloid leukaemia) compared with their wild-type and the gold standard ETA’-hCFP, respectively [34, 223, 227]. Their targeted cytotoxic effects were corroborated by Yoon *et al*. (1999) which specifically killed EGFR-expressing cells using EGFR-Ang fusion proteins using IC_50_ concentrations of 12.5–45 nM [223, 227].

### Microtubule-associated protein tau

Microtubules are critical structures in the process of cell division; they allow the alignment of chromosomes along the metaphase plate, before chromatids are pulled towards opposite poles during anaphase [212]. This process is tightly regulated through an evolutionary conserved checkpoint known as the spindle assembly checkpoint (SAC) [228]. Anti-mitotic drugs, most specifically the microtubule-targeting agents (MTAs), disturb normal spindle formation, activating SAC, resulting in cell cycle arrest, and cell death [229]. However, despite their potency and versatile application in oncology, MTAs lack specificity towards cancer cells and their repeated usage gives rise to the phenomenon of drug resistance [32, 230]. In view of addressing these challenges, a human anti-mitotic protein (known as MAP tau) was identified, showing similar activities as MTAs and allowing the development of potential hCFPs [231–234].

MAP tau belongs to a family of proteins (the microtubule-associated proteins), which regulate the stability of microtubules [211, 234, 235]. More explicitly, MAP tau binds to tubulin in a longitudinal fashion, causing the bridging of tubulin interfaces and hampering the shrinking phase of microtubule dynamics [32]. Given this indispensable role, the first MAP tau-based hCFP was thus engineered, consisting of an anti-EGFR scFv genetically fused to MAP tau isoform 3 [211]. To exclude the risk of neurodegenerative disorders, MAP tau-based hCFPs were deliberately designed with the vital phosphorylation sites of tau (S156 and S204) removed [211]. Moreover, the highly selective nature of the antibody fragment limits permeability through the blood–brain barrier and avoids the accumulation of MAP tau in the brain [211, 236].

Anti-EGFR (scFv)-MAP tau demonstrates specific cytotoxicity towards cells that express its target receptor, and no activity towards EGFR-negative HEK293 cells [32, 211]. However, the efficacy of this effector protein is highly dependent on cell proliferation, making rapidly dividing cancer cells as the target of choice for MAP tau-based hCFPs [211]. Moreover, this fusion protein showed increased tolerance in xenograft mice tumor models as compared to the PE-based control [211, 234, 237]. Like recombinant ITs, hCFPs avoid the need for complex chemistry processes and can be produced in large quantities in a one-step fermentation process [211]. Nonetheless, despite their potential clinical value, ease of manufacture and suitability for the development of patient-tailored therapies, their escape mechanism from the endosomes to the cytosol remains unclear [234, 237, 238]. Further investigation is therefore needed to enhance the preclinical therapeutic efficacy of these anti-cancer agents.

## CONCLUSIONS AND FUTURE DIRECTIONS

**Figure 5:**
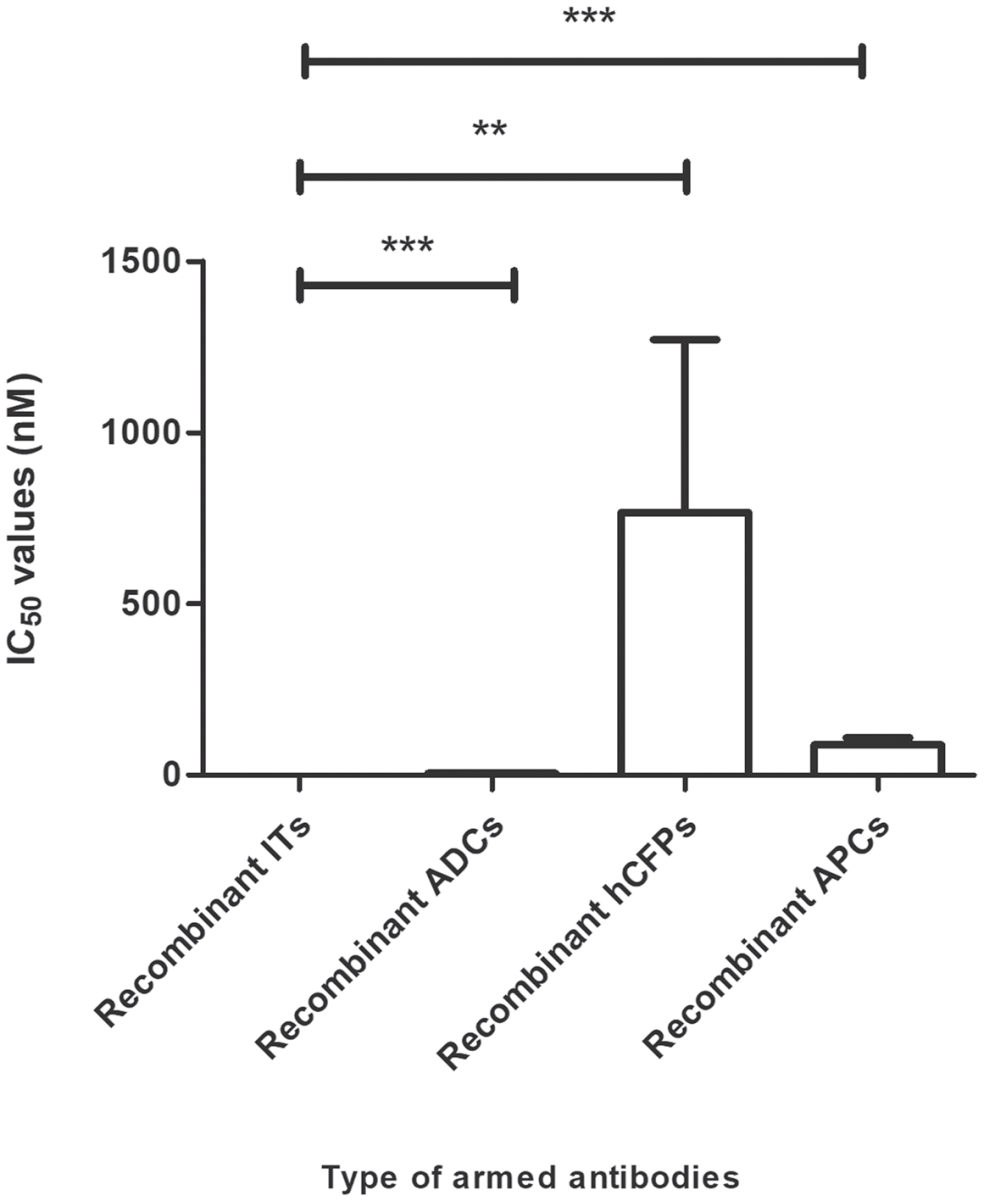
Comparative analysis of the efficacy of immunoconjugates currently in development. Based on publicly sourced IC_50_ values, recombinant ITs are highly cytotoxic agents. Student’s *t*-tests were performed to determine statistical differences (*p* < 0.05) between recombinant ITs and the other classes of therapeutics. Results reinforced the fact that recombinant ITs display higher cytotoxicities than recombinant ADCs (*p* < 0.0001), recombinant hCFPs (*p* < 0.005) or recombinant APCs (*p* < 0.001).

Cancer treatment has been revolutionized by antibody-based therapies that allow for specific targeting of diseased cells. However, the early promise of naked mAbs was hampered by the low success rates (15%) from Phase I to FDA approval [234]. The advent of genetic engineering ensured the evolution of therapeutic mAbs to yield molecules with reduced immunogenicity, increased half-life and enhanced ability to recruit immune effector responses. Among these, warhead-armed mAbs, are improved, highly potent cytotoxic compounds albeit, with a limited number currently approved for clinical use—attributed to insufficient, detailed knowledge on strategies on how to best deliver different payloads to their target intracellular compartments [239]. This is evident with EGFR-specific passive immunotherapeutics, whereby a preclinical comparison of the calculated average IC_50_ values ± standard deviations of the constructs described in the tables of this review are as follows: 0.133 ± 0.039 nM (recombinant ITs), 5.700 ± 1.446 nM (recombinant ADCs), 88.48 ± 21.13 nM (recombinant APCs), 766.7 ± 505.1 nM (recombinant hCFPs). These relative cytotoxic differences for the total number of constructs analyzed is more or less reflected for the Panitumumab-derived constructs showing the same principle results, while confirming the following efficiencies in selective cell killing; rIT > rADCs > rAPC > rhCFP (Figure 5).

Several decades of research ascribes this to differences in efficient delivery of the different types of warheads: for example, PE possesses functional domains which facilitate retrograde transport from the Golgi to the endoplasmic reticulum and improve translocation into the cytosol, while avoiding lysosomal degradation [240]. Furthermore, the catalytic capability of the warhead is another important factor, whereby the numbers of molecules needed to induce apoptosis, influences efficacy, favoring payloads where only a few molecules reach their target compartments yet induce efficient cell killing [241]. Thus, the next generation of immunoconjugates will necessitate alterations to the antibody and/or the cytotoxic moieties and will likely be dependent on target receptor densities, valency of the constructs, efficiency of receptor mediated uptake and subcellular delivery of warheads to their compartment of action. To this end, some progress has ensued, with the engineering of bispecific antibodies and protein translocation domains flanked with cleavable adapters to allow efficient internalization and transport of lethal warheads into the cytosol, respectively [242]. Additionally, the introduction of supercomputing tools to study enzyme-substrate interactions [33, 159], and modelling simulations that measure cellular processing parameters including binding, internalization, trafficking, and drug release/accumulation will undoubtedly foster the development of next-generation highly efficient apoptosis-inducing molecules [32]. The future curative potential of immunoconjugates will rely on emerging multimodality combinatorial approaches that explore non-overlapping mechanisms of action and toxicity profiles, resulting in synergistic efficacy. Continued optimization of antibody-mediated therapeutics and the evolving era of personalized treatment ensures a diversified immunotherapy armamentarium to combat cancer and improve patient outcomes.
